# The Economic Burden Attributable to a Child’s Inpatient Admission for Diarrheal Disease in Rwanda

**DOI:** 10.1371/journal.pone.0149805

**Published:** 2016-02-22

**Authors:** Fidele Ngabo, Mercy Mvundura, Lauren Gazley, Maurice Gatera, Celse Rugambwa, Eugene Kayonga, Yvette Tuyishime, Jeanne Niyibaho, Jason M. Mwenda, Philippe Donnen, Philippe Lepage, Agnes Binagwaho, Deborah Atherly

**Affiliations:** 1 Université Libre de Bruxelles, Ecole de Santé Publique, Brussels, Belgium; 2 Ministry of Health, Rwanda Biomedical Center, Kigali, Rwanda; 3 PATH, Devices and Tools Global Program, Seattle 98121, Washington, United States of America; 4 PATH, Vaccine Access and Delivery Global Program, Seattle 98121, Washington, United States of America; 5 World Health Organization, Rwanda Country Office, Kigali, Rwanda; 6 World Health Organization, Regional Office for Africa, Brazzaville, Republic of Congo; 7 Hôpital Universitaire des Enfants Reine Fabiola, Université Libre de Bruxelles, Brussels, Belgium; 8 Ministry of Health, Kigali, Rwanda; 9 Harvard Medical School, Boston, Massachusetts, United States of America; 10 Geisel School of Medicine, Dartmouth, Hanover, New Hampshire, United States of America; London School of Hygiene and Tropical Medicine, UNITED KINGDOM

## Abstract

**Background:**

Diarrhea is one of the leading causes of childhood morbidity and mortality. Hospitalization for diarrhea can pose a significant burden to health systems and households. The objective of this study was to estimate the economic burden attributable to hospitalization for diarrhea among children less than five years old in Rwanda. These data can be used by decision-makers to assess the impact of interventions that reduce diarrhea morbidity, including rotavirus vaccine introduction.

**Methods:**

This was a prospective costing study where medical records and hospital bills for children admitted with diarrhea at three hospitals were collected to estimate resource use and costs. Hospital length of stay was calculated from medical records. Costs incurred during the hospitalization were abstracted from the hospital bills. Interviews with the child’s caregivers provided data to estimate household costs which included transport costs and lost income. The portion of medical costs borne by insurance and household were reported separately. Annual economic burden before and after rotavirus vaccine introduction was estimated by multiplying the reported number of diarrhea hospitalizations in public health centers and district hospitals by the estimated economic burden per hospitalization. All costs are presented in 2014 US$.

**Results:**

Costs for 203 children were analyzed. Approximately 93% of the children had health insurance coverage. Average hospital length of stay was 5.3 ± 3.9 days. Average medical costs for each child for the illness resulting in a hospitalization were $44.22 ± $23.74 and the total economic burden was $101, of which 65% was borne by the household. For households in the lowest income quintile, the household costs were 110% of their monthly income. The annual economic burden to Rwanda attributable to diarrhea hospitalizations ranged from $1.3 million to $1.7 million before rotavirus vaccine introduction.

**Conclusion:**

Households often bear the largest share of the economic burden attributable to diarrhea hospitalization and the burden can be substantial, especially for households in the lowest income quintile.

## 1.0 Introduction

Diarrhea is the second leading cause of death for children less than five years old, causing approximately 751,000 childhood deaths in 2010 [1]. A major cause of diarrheal disease is rotavirus and it accounts for approximately 192,700 deaths annually among children less than five years old [2]. The global burden of diarrheal disease and deaths has been disproportionate between poor and rich countries, with about 50% of the deaths occurring in the African region [2]. The region also experiences about 9.6 million severe diarrhea episodes annually in children less than five years old [2]. Estimates for 2010 show that approximately 38,000 Rwandan children less than five years old died from all causes and one of the leading causes of death was diarrhea, accounting for 12% of the deaths [1]. In addition, a recent study estimated that from 2009 to 2011 there were between 13,153 and 17,254 non-bloody diarrhea hospitalizations in Rwanda [3].

Health insurance helps reduce the economic burden to households that can arise due to a household member’s illness. Health insurance coverage in Rwanda is high with over 90% of the population subscribing to Mutuelle de Santé, a community-based health insurance scheme (CBHIS). Insurance for the remaining segment of the population is covered by Rwandaise d’Assurance Maladie (RAMA) for civil servants, Military Medical Insurance (MMI) for members of the military and Caisse Sociale du Rwanda (CSR) for private sector employees [4].

Even with health insurance, households still face an economic burden if the child is hospitalized, since health insurance does not cover 100% of the medical costs and households still face other costs (e.g., transport costs and productivity losses). Few studies have estimated the economic burden of each inpatient admission for diarrhea borne by the health system and by households in African countries. In Ghana, the direct medical costs (e.g., hospital stay, diagnostics, medications, and medical staff time) were estimated to range from $65 to $97 [5], while a study conducted in South Africa estimated that the average direct medical costs ranged from $937 to $1140, and the average household costs were $16 [6]. Household costs include out of pocket (OOP) medical expenses, transport costs and lost wages. In Kenya, household costs were estimated at $19.46 [7]. Another study in Kenya estimated that the direct medical and household costs were $100 combined [8].

Rotavirus vaccination holds much promise for substantially decreasing the global health and economic burden of diarrhea [9,10]. In May 2012, Rwanda introduced the pentavalent rotavirus vaccine (RotaTeq^®^, Merck Vaccines, Whitehouse Station, New Jersey) into the routine Expanded Program on Immunization (EPI). The last-dose national coverage rates for rotavirus vaccine were reported to be 99% in 2012 and 100% in 2013 [11]. As part of the post vaccine introduction activities, several evaluations are being undertaken to help determine the impact and effectiveness of rotavirus vaccine.

As cited above, there have been few studies in Africa assessing the economic burden of diarrhea [5–8], and little is known about the economic burden of diarrheal disease in Rwanda. Our study serves to fill this knowledge gap by estimating the economic burden attributable to a diarrhea illness resulting in a hospitalization among children less than five years old and specifically the costs borne by health insurance payers and households. We also compared the estimated costs per diarrhea illness that resulted in a hospitalization for each study site to the estimated costs for the pooled study sample. In addition, we estimated the total economic burden associated with diarrhea hospitalizations before and after the rotavirus vaccine introduction, for the admissions reported by all public health centers and district hospitals in Rwanda. The analysis presented in this paper has been conducted as part of the post vaccine introduction studies and will be used to inform policymakers regarding the value of the rotavirus vaccination program.

## 2.0 Materials and Methods

### 2.1 Rwanda health insurance system

The majority of Rwandans are enrolled in the CBHIS. Government and donor subsidies help finance the enrollment of poor and vulnerable populations which comprise 25% of the CBHIS subscribers. Annual membership costs for CBHIS range from 3,000 Rwandan Francs (RWF) which is approximately 4 United States dollars, (US$, hereafter $) to 7,000 RWF (approximately $10). For the RAMA insurance for civil servants, the contribution rate is 15% of the basic salary of which 7.5% is paid by the employer and 7.5% by the employee. For MMI insurance for military families the contribution rate is 22.5% of gross salary, of which 17.5% is paid by the government and 5% by each military staff member. These other health insurance schemes donate a percentage of their annual earnings to CBHIS in order to sustain its activities [4].

Under the various health insurance schemes, health services can be obtained from primary health facilities as well as from district and national hospitals, and from accredited private providers. Except in the case of emergency situations, the referral system must be followed for the medical expense to be covered by insurance. The health insurance package of benefits covers outpatient and inpatient care, maternity care, essential drugs, medical imaging, and laboratory tests. Households also contribute copayments and the level of copayment varies by specific service. For example, there is no copayment for assisted birth delivery and surgery. For other services the households pay either 10% or 15% while insurance pays 90% or 85% of the cost, respectively; this varies by the type of health insurance scheme. The Ministry of Health compiles a schedule of tariffs for each medical service, drug, and consumable which are then used by the providers to charge for services provided [4].

The financial bill after a hospitalization is itemized for each type of medical service, drug, or consumable the patient received. Each line item in the bill includes the date, code in the tariff schedule, the type of service/drug/consumable received, the quantity received, the unit price, and the total cost for the line item. The bill also includes total costs to be paid by insurance and the OOP medical costs for the patient. Formal contracts are established between the health insurance schemes and the accredited public and private health care providers. Health care providers are reimbursed after they send a claim to the health insurance company [4].

### 2.2 Type of study and data collection methods

This was a prospective, cross-sectional costing study. Data were collected through a review of patients’ medical and financial records, which were obtained from each of the hospitals involved in this study and through household interviews with the caregivers of the patients enrolled in the study. Sample questionnaires contained in the World Health Organization’s guidelines for estimating the economic burden of diarrhea disease [12] were modified to suit the country setting. These questionnaires were used to record the data obtained from the medical and financial records and to conduct the household interviews.

### 2.3 Study sites

A purposive sampling approach was used to select the hospitals included in the study. The three hospitals included in the study were: Muhima Hospital, a public district hospital located in Kigali, the capital city of Rwanda, which serves an urban population of approximately 285,000; Rwamagana Hospital, a public district hospital located in the Eastern Province, which serves a rural population of approximately 310,000; and Kigali Teaching Hospital, a referral hospital also located in Kigali, which serves a population of approximately 1 million. These hospitals were selected because they were study sites for the other post vaccine introduction studies that were being undertaken.

### 2.4 Inclusion criteria

Children enrolled in our study met three inclusion criteria: the child was less than five years old and presented to one of the study sites with non-bloody diarrhea as the primary reason for consultation; the child was admitted into hospital and treated with oral rehydration or intravenous fluids; and the caregiver of the child consented to having the child included in the study and signed the informed consent form.

### 2.5 Sample size, ethics approval and data collection period

To inform sample selection, we used data on the number of non-bloody diarrhea hospitalizations which we obtained from the Rwanda Health Management Information System (HMIS). The HMIS is a health reporting portal and all public health centers and district hospitals in Rwanda submit monthly reports on specified health indicators into the HMIS. The HMIS does not include data from the national referral hospitals or private health facilities [3]. Using the data in the HMIS we estimated that there had been an annual average of 255 and 225 non-bloody diarrhea hospitalizations among children less than five years old between 2008 and 2011 at Rwamagana and Muhima District Hospitals, respectively. A registry count had estimated that there were 110 admissions at Kigali Teaching Hospital in 2011. Therefore, the samples sizes required to achieve a 10% level of precision with a 0.5 coefficient of variation were estimated to be 70 for Rwamagana hospital, 68 for Muhima hospital, and 52 for Kigali Teaching Hospital.

This study was approved by the Rwanda National Ethics Committee and the PATH Research Ethics Committee. Data collection occurred between November 2013 and June 2014.

### 2.6 Data collection and analysis

The study included three data collectors, one assigned to each of the study sites. Each study site also had a focal nurse who identified children eligible for the study and alerted the data collector about the admission of a child meeting the study’s enrollment criteria. The data collector’s first interaction with the child’s caregiver (parent or guardian) occurred while the child was still hospitalized. During this encounter the data collector explained the study to the caregiver and asked them if they wanted the child to be enrolled into the study. If the caregiver agreed, they had to provide written consent. The data collector then returned to the hospital after the child was discharged and extracted information from the child’s medical record. Information extracted from the medical record included: the date of admission and date of discharge, outcome on discharge, diagnoses made, diagnostic tests done, and medications administered while hospitalized and prescribed for use after discharge. The data collector also visited the hospital’s accounting department to collect information on financial bill for the hospital stay. The hospital financial bill was itemized, including information on services received, quantity and unit costs of resources used, and total charges. The data collector extracted the charges and grouped them into three categories: medications, diagnostic tests, and all other hospital charges (e.g., consultations, bed day costs, and consumables). These categories were clearly defined on the hospital bill.

The data collector followed up with the caregiver between 7 and 14 days after the child was discharged to interview them about the costs of care before the child was hospitalized and after the child was discharged. The data collectors visited most of the caregivers in their homes and conducted in person interviews, but a few caregivers were interviewed by phone. The caregiver reported the costs incurred for any medications, diagnostic tests, and consultations before and after the child’s hospitalization. They also collected household OOP costs incurred because of the child’s illness and obtained other socioeconomic information about the household. Household OOP costs included transport costs for the caregiver and child on the day the child was hospitalized and transport and accommodation costs for household members who visited the child while he/she was hospitalized. Information was also collected on the type of activities the caregiver would have been doing had the child not been hospitalized, their profession, daily or monthly income, and number of days of income lost because of taking care of the sick child. Visitor data, including their profession, number of days of income lost, and daily or monthly income was also collected. Data to characterize the household was obtained, including household monthly income, type of toilet facility, and source of drinking water.

All data were entered into a patient-level database in Excel (Microsoft. Redmond, WA, USA). Costs were entered in RWF and an exchange rate of 668 RFW per US$ was used to convert all costs to US$ [13]. Data analysis was done using Stata version 13 (Stata Corporation LP, College Station, TX, USA).

Excluded from the study were children who died, children whose caregiver absconded from the hospital without paying the medical bills, and children whose caregivers later chose to withdraw from the study after initial consent.

Direct medical costs incurred before, during, and after hospitalization for the diarrhea episode were calculated for each child as:

Direct medical costs = diagnostics tests costs+ medication costs + other hospital costs

Table 1 shows the unit prices of selected medical services, drugs, and consumables that were typically seen on the bills after a diarrhea hospitalization. The direct medical costs were categorized as direct medical costs paid for by insurance and direct medical costs borne by the households. The financial bills from the hospital admissions clearly separated the payers (the portion paid by insurance versus the patient’s responsibility).

**Table 1:** Unit costs in US$ for selected drugs, consumables and medical service used during a diarrhea admission based on the Rwanda tariff schedule.

	Unit	Unit price
**Drugs**		
Amoxicillin syrup 125mg	5ml	$0.68
Ampicillin injectable	1 vial—500mg	$0.12
Paracetamol 125g	5ml	$0.38
Ringer lactate	500ml	$0.98
Zinc sulphate 20mg	Each	$0.05
Oral rehydration solution	1 liter	$0.11
Vitamin A—200 000UI	1 tablet	$0.24
**Consumables**		$0.00
Laboratory request form	Each	$0.07
Catheter court IV UU 24G	1 piece	$0.31
Examination gloves	1 piece	$0.05
Syringe—5ml	1 piece	$0.07
General charges—patient file	Each	$0.15
General charges—medical prescription form	Each	$0.07
**Medical services, bed day costs, laboratory tests and others**		Tariff for Community Based Health Insurance Scheme
Medical consultation—first visit by a general doctor during hospitalization		$2.25
Medical consultation—subsequent visit during hospitalization by a general doctor		$0.45
Medical consultation—first visit by a specialist doctor during hospitalization		$2.02
Medical consultation—subsequent visit during hospitalization by a specialist doctor		$0.67
Medical care by a nurse		$0.45
Medical care by a nurse overnight or a weekend		$0.67
Medical care by nurses—administration of an intermuscular injection		$0.13
Medical care by nurses—administration of an intravenous injection		$0.18
Medical care by nurses—monitoring vital signs		$0.03
Bed day costs (in a large communal ward)		$0.72
Bed day costs (in a double room with outside bathroom)		$1.35
Bed day costs (in a single room with outside bathroom)		$2.25
Bed day costs (in a single room with inside bathroom)		$3.59
Laboratory test—hemoglobin		$0.57
Laboratory test—full blood count		$2.25
Laboratory test—glucose		$0.57
Ambulance per kilometer		$0.60

Some of the caregivers did not have bills for the outpatient care the child received before and after hospitalization. These caregivers self-reported the costs of care and we asked them to also report the amount they paid and the amount paid by insurance.

The direct non-medical costs borne by the household were estimated as:

Direct non−medical costs = round trip transport costs for the patient and caregiver + round trip transport costs for visitors from the child’s household + accommodation costs for visitors from the child’s household

Household income lost due to the child’s illness were estimated as:

$$Lost\;income=\sum_{i}^{n}\left(number\;of\;days\;of\;work\;lost_{i\;}X\;reported\;daily\;wage_{i}\right)$$

*where i* = *1,2,….n, where n is the number of household members with lost income*.

Household members reported either a monthly salary or a daily wage. In order to convert the monthly salaries into daily wages, we assumed that there were 22 work days per month and divided the monthly salary by 22 to estimate the daily wage rate.

Descriptive statistics to characterize the child and the child’s household were presented as the percentage for each study site. We also estimated the average length of stay (LOS) in hospital and the standard deviation. Mean, median and standard deviation of the costs were reported. We also calculated the 95% confidence intervals for the costs borne by insurance and households. Hypothesis tests (to test if the mean LOS and costs for each study site were different from the mean costs for the pooled data) were conducted using Students t-test, with p <0.05 showing significant difference for the two-tailed test. In addition, we calculated the household costs (the total of the OOP direct medical costs, transport costs and lost income) as a percentage of the household monthly income and reported these statistics by income quintile for our sample.

### 2.7 Estimating the annual economic burden of diarrhea hospitalizations

Using data on the annual number of diarrhea hospitalizations obtained from the HMIS [3], we estimated the annual economic burden associated with diarrhea hospitalization in each of the four years prior to the rotavirus vaccine introduction (2008 to 2011) and in 2014, after vaccine introduction. This economic burden represents the burden associated with admissions to the health centers and district hospitals since the HMIS data does not include data from the private health facilities or the national referral hospitals. Data quality assessments done show that all health centers and district hospitals report these diarrhea hospitalizations into the HMIS [3]. In estimating the annual economic burden across the years, we used the economic burden per diarrhea illness that resulted in a hospitalization that we estimated using the pooled data from the three study sites and assumed that this cost would not vary across the years. We calculated the economic burden borne by each payer (insurance and households).

The annual economic burden borne by the health insurance payers was estimated as:

$$Annual\;economic\;burden\;borne\;by\;health\;insurance_{t}=\;average\;direct\;medical\;costs\;borne\;by\;insurance\;per\;diarrhea\;illness\;resulting\;in\;a\;hospitalization\;\times \;number\;of\;diarrhea\;hospitalizations\;reported\;in\;the\;HMIS_{t}$$

The annual economic burden borne by the households was estimated as:

$$Annual\;economic\;burden\;borne\;by\;households_{t}=\;\left(average\;direct\;medical\;costs\;borne\;by\;households\;per\;diarrhea\;illness\;resulting\;in\;a\;hospitalization\;+\;average\;direct\;non\text{—}medical\;costs\;per\;diarrhea\;illness\;resulting\;in\;a\;hospitalization\;+\;average\;lost\;income\;per\;diarrhea\;illness\;resulting\;in\;a\;hospitalization\right)\;\times \;number\;of\;diarrhea\;hospitalizations\;reported\;in\;the\;HMIS_{t}$$

*Where t is the year for which the number of hospitalizations is reported*

The annual economic burden borne by insurance and households were then summed to estimate the annual economic burden for all payers for admissions to health centers and district hospitals. These annual economic burden calculations were also done using the lower and upper bounds of the 95% confidence intervals of the average costs per diarrhea illness resulting in a hospitalization.

## 3.0 Results

### 3.1 Study sample and descriptive statistics

A total of 214 children were enrolled in the study; the final sample used in the analysis was 203, as some children were discontinued from the study because they died (n = 5), they absconded from the hospital and therefore had incomplete medical or financial data (n = 5), or their caregivers chose to withdraw them from the study (n = 1). Enrollment targets were met for Muhima and Rwamagana District Hospitals but not for Kigali Teaching Hospital. The low enrollment number at Kigali Teaching Hospital was due to very low numbers of children presenting with diarrhea as this is a referral hospital, and the fact that sections of the pediatric unit were closed for renovations during part of the data collection period.

The sample description is shown in Table 2. Approximately 57% of the children were less than 1 year old and 93% of them had insurance coverage. The majority of the households (57%) obtained water from a shared community tap and they reported that they paid for OOP costs by cutting back on other household expenses.

**Table 2:** Patient and household characteristics.

	Muhima Hospital n = 109	Rwamagana Hospital n = 86	Kigali Teaching Hospital—CHUK n = 8	All sites n = 203
**Patient characteristics**				
*Gender*				
Female	45.0%	48.8%	25%	46%
*Age (months)*				
0–11	59.6%	53.5%	62.5%	57%
12–23	30.3%	33.7%	25.0%	32%
24–59	10.1%	12.8%	12.5%	11%
Mean age ± s.d. (months)	13.4 ± 9.9	14.6 ± 10.4	11.6 ± 10.7	13.9 ± 10.1
*Insurance coverage*				
Yes	88.9%	96.5%	100%	92.5%
**Household characteristics**				
*Sources of drinking water*				
Shared community tap	54.1%	63.5%	25.0%	57.1%
Tap to house	34.9%	22.4%	50.0%	30.1%
Open well	7.4%	0%	25.0%	4.9%
Lake or river or spring	0%	10.6%	0%	4.4%
Covered well	1.8%	3.5%	0%	2.5%
Borehole	1.8%	0%	0%	1.0%
*Type of toilet*				
Improved latrine	68.8%	45.3%	12.5%	56.7%
Pit latrine	11.9%	53.5%	25.0%	30.0%
Flush toilet	19.3%	1.2%	62.5%	13.3%
*Income*				
Reported household income per month: mean ± s.d. (US$)	$258 ± 175	$164 ± 165^a^	$346 ± 246^b^	$224 ± 180^c^
*Sources of income to pay for the OOO expenses*				
Cutting down on other expenses	90.8%	72.1%	62.5%	81.8%
Savings	0%	17.4%	25.0%	8.3%
Donations	7.4%	7.0%	0%	6.9%
Selling assets	0.9%	2.3%	0%	1.5%
Borrowing	0%	1.2%	12.5%	1%
Other	0.9%	0%	0%	0.5%

Not all respondents provided their monthly income:

^a^ n = 77;

^b^ n = 7;

^c^ n = 193;

Abbreviations: s.d. = standard deviation; OOP = Out of pocket expenses

Mothers were the main caregivers (96%) for the children during hospitalization and the majority of the caregivers (65%) reported that their highest level of education was primary school. The two most common activities by the caregivers were cultivating/farming (24%) and housework (22%).

About 70% of the children had been ill for 1 to 3 days before hospitalization and 80% of them had received care from a health center or community health worker before hospital admission (data not shown in tables). The mean LOS in the hospital was 5.3 days for the pooled sample and the median LOS was 4 days. Only the mean LOS at Kigali Teaching Hospital (2.5 days) was significantly different from the mean LOS for the pooled sample (p = 0.04), most likely because of the small sample from this hospital. Only 12 (6%) of the children received outpatient care after being discharged from the hospital.

### 3.2 Costs per diarrhea illness that resulted in hospitalization

For the 80% of children who sought care from a health center or community health worker before hospitalization, the average medical costs of this outpatient care were $6.12 ± $4.32 with the largest share of the costs being consultation fees. The average medical costs for the 12 children who sought care after being discharged were $3.94 ± $4.56. When averaged across the entire sample, the average medical costs for care received before and after hospitalization were $5.30 ± $4.62.

Table 3 presents the mean, standard deviation, and median costs associated with diarrhea illness, which includes the costs of the hospitalization and the costs for outpatient care before and after hospitalization (where the latter costs are reported above). Costs for diagnostic tests were estimated at $7.13 for the pooled sample; there were statistically significant differences between the pooled estimate and the estimates from each study site. The most common diagnostic tests ordered were full blood count (99.5% of the children), C-reactive protein tests (93%), and stool microscopy (85%). Based on the medical records, children admitted to Rwamagana Hospital also were more likely to get glucose tests (78% compared to 46% for the pooled sample) and microscopy (85% compared to 46% for the pooled sample). The cause of this variation was not explored as part of this study.

**Table 3:** Direct medical costs for the diarrhea illness in US$: mean ± standard deviation and median costs in parenthesis.

	Muhima Hospital n = 109	Rwamagana Hospital n = 86	Kigali Teaching Hospital—CHUK n = 8	All sites n = 203
Diagnostic tests	$4.66* ± 4.49 (2.79)	$8.85* ± 5.30 (7.94)	$22.40* ± 4.57 (22.92)	$7.13 ± 6.09 (4.70)
Medication	$16.24 ± 13.08 (13.13)	$12.77 ± 8.01 (10.77)	$16.71 ± 7.32 (18.80)	$14.79 ± 11.11 (12.75)
Other (consultations, bed day and consumables)	$21.35 ± 15.40 (17.46)	$22.52 ± 20.40 (15.52)	$18.99 ± 8.25 (17.85)	$21.75 ± 17.47 (16.86)
Total direct medical costs	$42.86 ± 22.41 (37.84)	$44.64 ± 25.80 (36.35)	$58.12 ± 13.15 (60.50)	$44.22 ± 23.74 (37.68)
Amount paid by insurance	$31.69 ± 22.59 (29.73)	$37.42 ± 23.32 (32.30)	$49.75 ± 10.46 (54.00)	$34.83 ± 22.86 (31.70)
OOP	$11.26 ± 17.69 (4.42)	$7.25 ± 11.09 (4.60)	$8.36 ± 4.98 (6.58)	$9.45± 14.96 (4.55)

* signifies a significant difference from the mean for all sites at 5% level of significance for a 2 tailed test

Medication costs were estimated at $14.79 for the pooled sample; there were no significant differences in the costs for the individual sites compared to the pooled data. The most common medications prescribed to each child in addition to rehydration solution were vitamins (64%), and antibiotics (62%). Other costs, including consultations, bed day costs, and consumables, were the largest share of costs (almost 50% of total direct medical costs).

Total direct medical costs for the diarrhea illness were estimated at an average of $44.22 ± $23.74 (median $37.68) for the pooled sample. There were no significant differences in the total direct medical costs for each site compared to average costs for the pooled sample. However, the median costs for Kigali Teaching Hospital were almost $20 higher than median costs for the other two hospitals, despite the shorter length of stay for children admitted to this hospital. Insurance covered almost 80% of the costs, and median OOP direct medical costs ranged from $4.42 to $6.58 per household across the three hospitals.

Table 4 shows the direct non-medical costs and lost income. None of the visitors reported incurring any hotel or lodging costs on their trips to visit the child while hospitalized. The most common modes of transport to the hospital for the child and caregiver were walking (29%) and riding the bus (26%). Average transport costs for the caregiver and child were estimated at $2.06 ± $2.56, and median total transport costs for all visitors were approximately $6 per admission for the pooled sample. The average household lost income due to the child’s illness was approximately $42 for the pooled sample. None of the estimated average transport or lost income costs from each site were statistically different from the pooled average. The transport costs for visitors and household lost income for children admitted to Kigali Teaching Hospital were lower than for the other sites, most likely due to the shorter LOS.

**Table 4:** Direct non-medical costs and lost income in US$: mean ± standard deviation and median costs in parenthesis.

	Muhima Hospital n = 109	Rwamagana Hospital n = 86	Kigali Teaching Hospital—CHUK n = 8	All sites n = 203
Transport costs for patient and caregiver	$1.92 ± 2.59 (0.90)	$2.14 ± 2.54 (1.50)	$3.21 ± 2.34 (2.77)	$2.06 ± 2.56 (1.20)
Transport costs for visitors	$12.41 ± 26.08 (5.09)	$13.84 ± 16.61 (8.98)	$6.72 ± 4.33 (5.98)	$12.79 ± 21.79 (5.99)
Lost income	$40.85 ± 50.42 (29.94)	$44.08 ± 59.72 (26.95)	$29.89 ± 52.63 (11.90)	$41.78 ± 54.44 (26.95)

**Fig 1:**
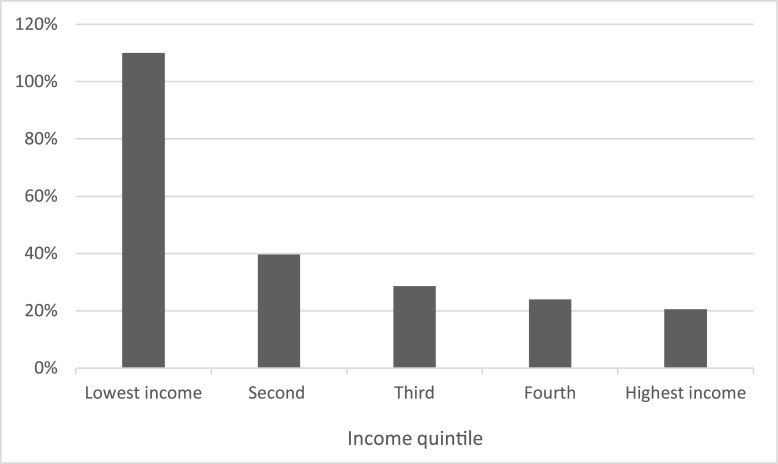
Household costs for the diarrhea illness resulting in hospitalization as a percentage of household monthly income by income quintile.

For each diarrhea illness that resulted in a hospitalization, we estimated the economic burden to be $101 per illness, which includes $44.22 in direct medical costs, $14.85 in direct non-medical costs and $41.78 in lost income. Insurance bore $34.83 (95% confidence interval: $31.67 to $37.99) or approximately 80% of the direct medical cost while households bore the remaining 20% of the direct medical costs. When including all the costs that are borne by the households we found that the households bore $66.08 (95% confidence interval: $53.26 to $78.92) which was 65% of the economic burden per diarrhea illness that resulted in a hospitalization. Fig 1 shows that for households in the lowest income quintile, the household costs per diarrhea illness averaged 110% of the reported monthly income, while for the other four income quintiles, the household costs as a percentage of monthly income were much lower, ranging from 40% in the second income quintile to 21% for the highest income quintile.

### 3.3 Estimated annual economic burden

In the four years prior to rotavirus vaccine introduction, there were between 13,153 and 17,254 diarrhea admissions in public health centers and district hospitals in Rwanda. The estimated annual total economic burden attributable to these diarrhea admissions ranged from approximately $1.3 million to $1.7 million, when using the average costs (Table 5). Unpublished data from the HMIS also shows that in 2014, after rotavirus vaccine introduction, there were 10,474 non-bloody hospitalizations, resulting in an estimated reduction in the total economic burden ranging from 20% to 40% compared to the four years prior to vaccine introduction. Although the introduction of the rotavirus vaccine was the most significant new intervention against diarrheal disease implemented during this period, other factors may also have contributed to the reduction in the economic burden.

**Table 5:** Estimated economic burden of non-bloody diarrhea hospitalizations from 2008–2011 and in 2014 in US$ and percentage difference in the economic burden compared to 2014 burden.

Year	Number of admissions	Direct medical costs borne by insurance	Household costs (direct medical, direct non-medical, and lost income)	Total economic burden	% difference compared to the average economic burden estimated for 2014
2008	15,928	$554,772 ($504,440 –$605,105)*	$1,052,522 ($848,325 –$1,257,038)*	$1,607,294 ($1,352,765 –$1,862,142)*	34%
2009	17,254	$600,957 ($546,434 –$655,479)*	$1,140,144 ($918,948 –$1,361,686)*	$1,741,101 ($1,465,382 –$2,017,165)*	39%
2010	16,412	$571,630 ($519,768 –$623,492)*	$1,084,505 ($874,103 –$1,295,235)*	$1,656,135 ($1,393,871 –$1,918,727)*	36%
2011	13,153	$458,119 ($416,556 –$499,682)*	$869,150 ($700,529 –$1,038,035)*	$1,327,269 ($1,117,084 –$1,537,717)*	20%
2014	10,474	$364,809 ($331,712 –$397,907)*	$692,122 ($557,845 –$826,608)*	$1,056,931 ($889,557 –$1,224,515)*	

*In parenthesis is the economic burden estimated using the lower and upper bounds of the 95% confidence interval of the costs per hospitalization

## 4.0 Discussion

Although previous studies have also evaluated the economic burden attributable to diarrhea hospitalization [5–8, 14–19], our study was the first assessment done in Rwanda and it adds to the literature on the economic burden of diarrhea in Africa. Our study estimated that the average LOS for a child hospitalized with diarrhea was five days, similar to what was reported by other studies [6,7,14]. We also found that for each diarrhea illness which results in a hospitalization, the total economic burden averages $101 per Rwandan child admitted. These estimates are similar to those reported in other African countries, specifically for studies done in Kenya and Ghana [5,8]. Direct medical costs associated with a diarrheal illness that results in a hospitalization were similar across the three hospitals in our sample, averaging $44.22 per illness. The largest driver (49%) of direct medical costs were other hospital costs (consultation, bed day costs, and consumables), which were similar at each hospital despite that children cared for at Kigali Teaching Hospital stayed on average of 2.5 fewer days than children hospitalized at Rwamagana Hospital and Muhima Hospital. The total direct medical costs estimated for Kigali Teaching Hospital were the same as those for the other two hospitals, despite the shortened LOS at Kigali Teaching Hospital. This primarily stemmed from increased costs for diagnostics tests, which were three times greater for Kigali Teaching Hospital when compared to the pooled average.

Most Rwandans have insurance (93%), which covered approximately 80% of direct medical costs for a diarrhea illness that resulted in a hospitalization, leaving households responsible for approximately 20% of these costs. The economic burden resulting from a child’s diarrhea illness which results in hospitalization accounts for between 21% and 110% of the household’s monthly income, with the largest burden falling on the households in the lowest income quintile. Therefore, these costs can be substantial especially for low income households. Other studies have characterized the heavy economic burden of diarrheal disease in low-income households [9,14, 20–21].

Before rotavirus vaccine introduction, hospital admissions due to non-bloody diarrhea among children less than five years old had cost Rwanda an annual estimated amount of $1,327,269 or the equivalent of $0.74 per child less than five years old using 2010 population data [22]. The majority (65%) of these costs were borne by households with the largest share (63%) of household costs attributed to lost income. In the period after vaccine introduction, the number of non-bloody diarrhea hospitalizations fell, and the estimated economic burden fell by approximately 20% to 40%. However, the decline in the hospitalizations and economic burden cannot be attributed solely to vaccine introduction, because, in part, non-bloody diarrhea hospitalizations had already been declining even before the vaccine was introduced [3] because of other initiatives such as better community case management of diarrhea and hygiene improvement.

This study found that the total cost, including direct medical costs, indirect medical costs, and lost income, associated with a diarrhea illness that results in a hospitalizations is similar (no different than the pooled average) across rural, urban, and referral district hospitals. Therefore, this economic cost data could be extrapolated to all diarrheal hospitalizations at referral hospitals and used in models to project costs averted as a result of rotavirus vaccine introduction as well as used as an input in cost-effectiveness studies.

This study also highlights the value of a well-functioning health insurance system. The availability of financial bills for each patient in our sample reduced or eliminated the likelihood of our study underestimating the direct medical costs, a risk that can arise when researchers have to abstract the quantities of resources used in each hospitalization, from paper medical records, to estimate the costs. These paper medical records can be incomplete or illegible, which then becomes challenging for researchers to account for all the resources used in the care of the patient.

This study has several limitations. The direct medical costs reported in this study may underestimate the total costs of care for children with diarrhea because health services are subsidized by the Government of Rwanda, and therefore the data reported in the financial bills may not reflect the full cost of care for each diarrhea case. Also, the study did not estimate facility overhead costs or infrastructure costs, which may not be included in the financial bills, although some of these costs may be included in the bed-day costs. In addition, this study did not consider costs associated with hospitalizations at health clinics, which according to a prior study accounted for 60% to 72% of diarrhea-related hospitalizations in Rwanda [3]. If the costs of hospitalization at these health clinics are lower than those at the district hospitals, we have overestimated the economic burden of hospitalized non-bloody diarrhea to the country. Future studies should include these health clinics as study sites. The economic burden estimates only include the admissions reported by public health facilities and district hospitals, and do not include admissions to private hospitals or the national referral hospitals. Therefore the total national economic burden of diarrhea is likely underestimated. We also encountered challenges with enrollment into the study at Kigali Teaching Hospital and were not able to meet the sample size target. The results from this site may not reflect the true LOS or the costs of care for a child admitted to this tertiary hospital for diarrhea. Future studies should aim to improve on the estimates we have reported at this hospital. Another limitation of our study is its focus only on the economic burden attributable to inpatient admissions. However, it is important to note that total economic burden due to diarrheal illnesses is significantly more than the costs solely associated with hospitalized cases since it has been estimated that there are approximately 10 to 12 outpatient visits for every inpatient diarrhea admission [9,10]. Future studies should aim to estimate the burden attributable to outpatient visits.

In conclusion, children with diarrhea were hospitalized for an average of five days. The economic burden per diarrhea illness that resulted in a hospitalization was $101 and 65% of this cost was borne by households. All the costs we estimated did not significantly differ across the three hospitals included in our study. The average annual economic burden to Rwanda from these hospitalizations ranged from $1.3 million to $1.7 million for the period just prior to the introduction of the rotavirus vaccine in the national immunization program, and declined by approximately 20% to 40% in 2014, 2 years after vaccine introduction, even though the entire reduction cannot be attributable to the vaccine introduction.
